# How different aspects of plasma treated liquids (PTLs) influence their antimicrobial properties

**DOI:** 10.1038/s41598-025-14338-9

**Published:** 2025-09-29

**Authors:** Kristína Trebulová, Kamila Klementová, Svatava Bednaříková, Daniela Dofková, František Krčma, Ivana Paličková, Miroslava Barančeková, Alois Čížek, Zdenka Kozáková

**Affiliations:** 1 Faculty of Chemistry, Brno University of Technology, Purkyňova 464, Brno, 612 00 Czech Republic; 2 University of Veterinary Sciences Brno, Palackého třída 1946/1, Brno, 612 42 Czech Republic

**Keywords:** Cold plasma, Plasma medicine, Antimicrobial efficacy, Plasma treated liquids, Indirect plasma treatment, Biophysics, Chemical biology, Microbiology, Health care, Physics

## Abstract

This work studies the decontamination efficacy of different plasma treated liquids (PTLs) on bacteria from the genera *Staphylococcus* and *Pseudomonas*, both commonly associated with various infections. Clinical isolates and reference strains were used, to ensure the relevance to real-life applications. Bacterial suspensions were exposed to studied PTLs and to different comparative solutions that help dissect the mechanisms behind the observed antimicrobial effects. These comparative solutions comprised standard solutions of major reactive species (hydrogen peroxide, nitrites, nitrates) typically found in PTLs, solutions simulating the chemical composition of PTLs, and solutions adjusted to different pH levels to isolate the role of acidity in bacterial inactivation. The antimicrobial effects of studied solutions were examined at various contact times from 10 min up to 24 h. This allowed for a comprehensive understanding of both immediate antimicrobial effects and the persistence of PTL´s activity over time. The findings of this research demonstrate a superior antimicrobial efficacy of plasma treated liquids compared to the other studied solutions. Neither the individual standard solutions of reactive species, the solutions simulating the chemical composition of PTLs, nor pH-adjusted solutions were able to match the antimicrobial efficacy of the tested PTLs. Although it has been found that for some bacterial species, pH of the PTL may play a key role in the decontamination efficacy. The study also shows that different PTLs vary in their antimicrobial efficacy, depending on the specific formulation and the type of targeted microbial species. These differences in bacterial response may be influenced by factors such as cell wall structure, antioxidant capacity, and pH tolerance. In conclusion, this work supports the potential of indirect cold plasma treatment (via PTLs) for antimicrobial purposes. It highlights the complex interplay of factors involved in microbial inactivation and offers deeper insight into the differing responses of gram-negative and gram‑positive bacterial species to various PTLs. Furthermore, the study provides an overview of the antimicrobial effects of individual components present in PTLs across a wide range of concentrations and pH conditions. This may help other researchers compare the efficacy of different antimicrobial agents and explore potential mechanisms of inhibition.

## Introduction

With the ever-increasing antimicrobial resistance, the efficacy of the conventional treatment methods rapidly decreases^1^. Thus, there arises an urge to find alternative treatments for many diseases in human and veterinary medicine. In the past few decades, cold atmospheric-pressure plasma (CAP) has seen a potential rise in a variety of fields including medicine^2,3^. The effects of CAP are associated with different aspects of plasma such as the generation of reactive species, electrons, ions, electromagnetic field and photons of various wavelengths including UV and VUV. Their synergistic effect makes CAP a unique technology with many possible applications in the medical field^4^. However, it is not always possible to use the gaseous plasma, which is why an interest in so-called plasma treated liquids arises^5,6^.

Plasma treated liquids (PTLs) are liquids that were exposed to plasma, in this study to the cold atmospheric-pressure plasma (CAP). This treatment is sometimes referred to as an indirect plasma treatment^7^ because a secondary agent (usually liquid), is plasma treated and then put into contact with the object of attention. The interaction of plasma with the liquid induces formation of new molecules leading to chemical and physical changes of the treated liquid. All the different attributes of plasma including the reactive oxygen and nitrogen species (RONS), electric and magnetic field, UV and VUV photons, electrons, etc. interact with the liquid resulting in a cascade of different processes^7–9^.

One of these processes is the transport of reactive species (RONS) from the gaseous plasma to the liquid. The reactive oxygen species (ROS) include, e.g., hydroxyl radicals, atomic oxygen, hydrogen peroxide, ozone, etc., while reactive nitrogen species (RNS) include mostly derivatives of nitrogen oxide such as nitrites, nitrates, and peroxynitrites. The RONS have different characteristics (e.g., hydrophilicity, lifetime), that influence which particles are preferentially transferred into the liquid and in what amounts. The presence of reactive species and the electromagnetic field induce various chemical reactions in the liquid or at the gas-liquid interface leading to the formation of secondary active particles^10^. The most prevalent reactive species detected in the PTLs are hydrogen peroxide (H_2_O_2_), nitrites (NO_2_⁻) and nitrates (NO₃⁻). These are the long-lived particles that are highly soluble in liquids, in greater quantities compared to more hydrophobic reactive species like ozone^11^.

Each reactive species plays a distinct role in contributing to antimicrobial efficacy. Hydrogen peroxide (H_2_O_2_) is capable of diffusing into bacterial cells, where it induces oxidative stress through the formation of hydroxyl radicals via Fenton-like reactions, damages DNA and proteins, and disrupts membrane integrity. Hydrogen peroxide (H_2_O_2_) along with superoxide (O_2_^−^) can disrupt essential enzymes (such as dehydratases) by targeting their iron-sulphur (Fe‑S) clusters. Oxidation of the exposed Fe‑S clusters leads to destabilization and potential disintegration resulting in a loss of enzymatic activity^12^. Nitrites (NO_2_⁻) play a dual role in the antimicrobial mechanisms of plasma-treated liquids^13^. First, under acidic conditions, NO_2_⁻ can be protonated to form nitrous acid (HNO_2_), which spontaneously decomposes to generate nitric oxide (NO). NO subsequently reacts with superoxide (O_2_⁻) to form peroxynitrite (ONOO⁻), a highly reactive oxidant capable of damaging bacterial DNA, proteins, and lipid membranes through nitration and oxidation reactions. This pathway is particularly relevant in low-pH PTLs, where such reactions are favoured^14^. Second, NO_2_⁻ also contributes to acidification of the medium, influencing both the intracellular pH homeostasis of bacteria and the chemical reactivity of other RONS, including H_2_O_2_ and NO-derived species. Additionally, nitrites may participate in direct nitration reactions that modify thiol groups and amines in bacterial proteins, further compromising cellular function^15^. While nitrate (NO₃⁻) itself is relatively inert and does not directly cause oxidative or nitrosative stress, it is considered an important storage and end‑product of nitrogen-based redox reactions. Its presence in PTLs may reflect prior formation of more reactive intermediates and could influence the long-term chemical stability of the liquid. It helps to modulate ionic strength and pH, potentially affecting the reactivity and lifetime of other RONS in the solution^16^. Its high solubility and stability may play a role in sustaining the antimicrobial properties of PTLs over time. Though not directly antimicrobial, nitrates contribute to the overall chemical environment in which RONS act.

The presence of the reactive species and their reactions change not only the composition of the treated liquid but also its conductivity, pH and the redox potential^7^. The acidic pH of PTLs is another factor contributing to their antimicrobial efficacy. pH levels significantly impact bacterial viability by influencing various cellular processes. Bacteria respond to pH changes by regulating gene expression and protein profiles. Oxidative stress (caused by RONS) can be amplified or reduced depending on the pH. A combination of oxidative stress and acidic pH can be particularly damaging to bacteria since, the acidic pH can increase the generation and activity of RONS^16,17^. For instance, in a phagosome within a macrophage, the low pH may promote the conversion of superoxide to hydrogen peroxide^18^. As mentioned above both hydrogen peroxide (H_2_O_2_) and superoxide (O_2_^−^) are critical molecules influencing the bacterial viability. This underscores the importance of RONS‑pH synergy in bacterial inactivation, particularly in indirect plasma applications like PTLs.

The properties of PTLs differ according to the type of plasma-liquid interaction, plasma source, working gas, treatment time and the liquid itself (composition, initial conductivity, pH, etc.). This makes each plasma treated liquid unique, especially when it comes to the major type and concentration of the long-lived RONS^7,19^.

There are several possible methods for treating liquids with plasma. The most frequently used option is direct plasma treatment. This means that the liquid is placed inside the electrical circuit. During the plasma-liquid interaction, UV radiation, electric field, heat and reactive species are generated^5^. The direct treatment of the liquid can be achieved by direct generation of plasma inside the liquid, by applying gaseous plasma to the surface of the liquid or by developing the so-called multiphase discharge^6^. Indirect treatment can be achieved using remote plasma systems introducing plasma activated gas into the liquid^20,21^. All the plasma systems mentioned above produce different compositions of the PTL. The discharges generated inside the liquid cause direct ionization of water molecules to hydroxyl radicals which leads to the intensive formation of hydrogen peroxide^22^. On the other hand, the formation of nitrites and nitrates is lower due to the limited amount of nitrogen and oxygen dissolved in the liquid. However, this factor can be improved by adding a gas flow through the plasma region. Contrary to the inside liquid systems, gaseous plasma interaction with the liquid provides higher amounts of nitrites and nitrates due to the increased formation of nitrogen oxides, ions, and radicals in the gas plasma^21,23^. Depending on the application, an appropriate system can be used to produce PTL with desired reactive species.

The plasma treated liquids can be used in different branches of industry. A very promising application is in agriculture^24^ where PTL not only decontaminates the seeds or plants^25,26^ but also helps to promote the seed germination and plant growth^27–29^. In medical sciences, the PTL sees its potential use in decontamination^30,31^ or plasma modification of various medical tools and utensils^32^. Further, it can be turned into plasma treated hydrogels^33^ that may be used for wound healing, cancer treatment or enhanced drug delivery^34^. All the various applications show promising results in a quickly growing field of plasma medicine. This study focuses on the decontamination effects of different PTLs for potential veterinary applications^35^. In cooperation with the Veterinary University in Brno, several bacterial strains that represent a global problem, not only in the field of veterinary medicine, were chosen for the experimental testing of PTLs. These strains include species from the *Staphylococci* and *Pseudomonas* genera, both the clinical isolates and reference strains. The testing of these two different groups of microorganisms renders the study more relevant to the real-life applications of PTLs.

## Materials and methods

### Plasma treated liquids

**Fig. 1:**
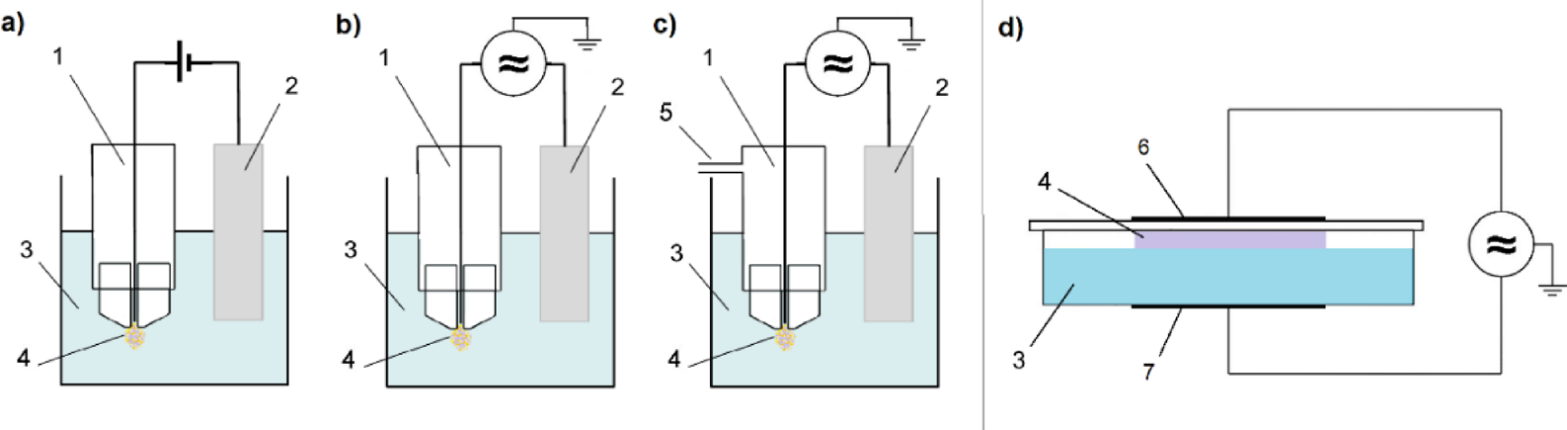
Schemes of plasma devices used for PTL preparation; plasma inside the liquid: (**a**) DC, (**b**) AC, (**c**) AC_air_, plasma above the liquid (**d**) (DBD); 1 - pinhole electrode, 2 - counter electrode, 3 - treated liquid (physiological solution), 4 - plasma, 5 - gas inlet, 6 - upper silver electrode on the ceramic plate, 7 - bottom graphite electrode on the Petri dish.

Plasma treated liquids (PTLs) were prepared by two different plasma systems generating plasma either above or inside the liquid. The plasma system based on the pinhole discharge, patented at Brno University of Technology, Faculty of Chemistry, Czech Republic^36^was used to make PTL by direct generation of plasma inside the liquid. This system was implemented in three modifications that can be seen in Fig. 1: (a) with the DC high voltage source (PTL_DC_)^37^(b) with the AC high voltage source without air bubbling (PTL_AC_), (c) with the AC high voltage source with air bubbling (PTL_ACair_)^29^. Additionally, the dielectric barrier discharge with the liquid electrode (PTL_DBD_)^20,38^ producing plasma above the liquid surface was used for the PTL preparation (Fig. 1d).

The plasma systems generating plasma inside the liquid (DC, AC or AC_air_) consisted of the main pinhole electrode (a tungsten wire in the glass holder ending with a glass or ceramic hollow head) with or without the additional gas input, the plate counter electrode made of alumina, and a vessel with 50 ml of the treated liquid (physiological solution; NaCl 9 g/l). PTL was prepared at the mean power of 40 ± 3 W (for both DC and AC high voltage source) applied for 1 min. In the case of additional air bubbling through the plasma region, an oxygen-nitrogen mixture in the ratio of 1:4 and the total flow rate of 0.5 l/min was used.

The above liquid plasma system (DBD) consisted of the bottom Petri dish containing 75 ml of the treated liquid with the outer graphite electrode, and the upper ceramic plate with the outer silver electrode. Both electrodes were connected to the high frequency source (Lifetech s.r.o., Czech Republic) operating at the peak-to-peak high voltage of 16 kV (frequency of 11 kHz). PTL from physiological solution (PS; NaCl 9 g/l) was prepared at the mean power of 36 ± 2 W applied for 5 min.

For each type of PTL, samples were taken to characterize the content of the selected long-lived species (hydrogen peroxide, nitrates, and nitrites). Colorimetric methods joined with the UV‑VIS spectrophotometry were employed for the analysis. Samples of the prepared PTL were mixed with appropriate colorimetric reagents and measured using a UV‑VIS spectrophotometer (Helios Omega, Unicam) at the appropriate wavelength of maximal absorption. Hydrogen peroxide was determined using a titanium reagent (titanium oxy-sulphate solution in 30% sulphuric acid, Merck) with the previous masking of nitrites by sodium azide. The resulting, yellow-coloured product was measured at the maximum wavelength of 407 nm. Nitrates and nitrites were determined using specific colorimetric kits (Tetra or Supelco) and measured at maximum wavelengths of 526 nm (nitrates) and 540 nm (nitrites), respectively. Additionally, the pH and specific conductivity of the prepared plasma treated liquids were measured. Compositions of all prepared PTLs including physical-chemical properties are discussed in the results section and summarized in Table-3.

Decontamination effects of PTLs prepared in above mentioned plasma systems (DC, AC, AC_air_, and DBD) were studied on selected microorganisms (*S. pseudintermedius*, *S. aureus*, *P. aeruginosa*). Further, the most promising PTLs plasma systems (DC and AC_air_) were compared with the effects of standard solutions of nitrates, nitrites and hydrogen peroxide and the solutions simulating the chemical compositions of the plasma treated liquids (artPTLs).

Standard solutions of nitrates (NO_3_^−^), nitrites (NO_2_^−^) and hydrogen peroxide (H_2_O_2_) were prepared in ranges of concentrations corresponding to their content in PTL up to the concentrations that, according to the literature^39–41^correspond to the minimum inhibitory concentrations (MIC). For the preparation of hydrogen peroxide standard solutions, 30% non‑stabilised hydrogen peroxide (Lach-Ner, s.r.o.) was used. Sodium nitrate (Lach-Ner, s.r.o.), and sodium nitrite (Lach­Ner, s.r.o.) were used for the preparation of nitrate and nitrite standard solutions. The antimicrobial effects of the prepared solutions were tested on the collection strains of *Staphylococcus aureus* and *Pseudomonas aeruginosa* (concentrations of standard solutions are shown in Table 1). All standard solutions were prepared in sterile distilled water.

**Table 1:** Concentrations of standard solutions prepared for antimicrobial tests on *S. aureus* and *P. Aeruginosa.*

H_2_O_2_ [mg∙l^− 1^]		NO_2_^−^ [mg∙l^− 1^]		NO_3_^−^ [mg∙l^− 1^]	
S. aureus	*P*. aeruginosa	S. aureus	*P*. aeruginosa	S. aureus	*P*. aeruginosa
2.5	15.0	1.5	1.5	25.0	25.0
15.0	100.0	10.0	5.0	50.0	50.0
25.0	1000.0	15.0	10.0	100.0	100.0
50.0	10000.0	100.0	100.0	500.0	500.0
100.0		500.0	500.0		
10000.0					

Solutions simulating the chemical composition of corresponding PTLs (later referred to as PTL alternatives or artPTL) were formulated to match the concentrations of hydrogen peroxide, nitrates and nitrites of the selected PTL. Standard solutions of individual reactive species were mixed and diluted in sterile physiological solution to the required concentration. The artPTLs were prepared for PTL_ACair_ and PTL_DC_, whose antimicrobial effects were evaluated as the most promising. The final concentrations of individual components simulating PTL_ACair_ (artAC_air_) and PTL_DC_ (artDC) solutions are shown in Table 2.

Standard solutions adjusted to different pH values (0.5, 2.5, 3.5, and 5.5) were prepared by diluting 65% nitric acid (Lach-Ner, s.r.o.) in sterile distilled water. The pH values of 3.5 and 5.5 were chosen according to the pH values of the used PTLs (3.5 for the PTL_ACair_, and 5.5 for the other PTLs). The lower pH values (0.5 and 2.5) were selected with the aim of finding a critical pH value for survival of the studied microorganisms. As a control, sterile PBS buffer was used, as in other measurement, with the pH value of 7.2 ± 0.2.

**Table 2:** Composition of solutions simulating PTL_DC_ (artDC), and PTL_AC_ (artAC).

	PTL_DC_	artDC	PTL_ACair_	artAC_air_
σ [mS∙cm^− 1^]	16.7 ± 0.5		16.5 ± 0.3	
pH [-]	5.2 ± 0.3	6.3 ± 0.3	3.5 ± 0.2	6.1 ± 0.3
H_2_O_2_ [mg∙l^− 1^]	16.0 ± 3.0	15.0	1.7 ± 0.3	1.5
NO_2_^−^ [mg∙l^− 1^]	0.2 ± 0.2	0.2	11.0 ± 3.0	10.0
NO_3_^−^ [mg∙l^− 1^]	25.0 ± 7.0	25.0	42.0 ± 3.0	45.0

### Microbiological part

For the testing of antimicrobial activity, four bacterial strains were used: two reference strains *Staphylococcus aureus* (ATCC 29213) and *Pseudomonas aeruginosa* (ATCC 27853) supplied by the Czech Collection of Microorganisms, Masaryk University Brno, and two clinical isolates *Staphylococcus pseudintermedius* and *Pseudomonas aeruginosa* supplied by the Veterinary University in Brno. The inclusion of both reference and clinical strains enhances the translational relevance of the study and allows assessment of antimicrobial efficacy under both standardized and real-world clinical conditions. These strains were selected due to their significant clinical relevance in veterinary medicine, particularly in the context of superficial infections such as *otitis externa*, *pyoderma*, and wound infections. *Staphylococcus aureus* ATCC 29,213 and *Pseudomonas aeruginosa* ATCC strains serve not only as quality control organisms in antimicrobial susceptibility testing, as per CLSI (Clinical and Laboratory Standards Institute) guidelines, but also as well-characterized models for studying resistance mechanisms in gram-positive and gram-negative pathogens, respectively. Clinical isolates, including *Staphylococcus pseudintermedius* and *Pseudomonas aeruginosa*, are frequently associated with infections in companion animals, especially dogs and cats. *S. pseudintermedius* is the leading cause of canine *pyoderma* and *otitis externa* and is increasingly associated with methicillin resistance and zoonotic transmission^42,43^. *P. aeruginosa* is a notorious pathogen in *otitis externa* and is also a common nosocomial pathogen exhibiting multidrug resistance and persistent biofilm formation^44^. Therefore, both species are of growing concern due to rising multidrug resistance and zoonotic potential^45^making them highly relevant targets for evaluating novel antimicrobial strategies such as plasma-treated liquids. Both clinical isolates in this study were isolated from dogs with *otitis externa*.

All microorganisms were cultured on the solid culture media appropriate for each genus: Columbia blood agar base with defibrinated sheep blood for the *Staphylococci* and MacConkey agar for the *Pseudomonas*.

The inoculum of the tested microbial strain was prepared in the corresponding medium and cultivated at the temperature of 37 °C for 24 h. The cultivation temperature was chosen as a standard temperature for the cultivation of mesophilic microorganisms and as a reference to a normal human body temperature. Even though the temperature in animal body may vary from the human body temperature. For example, the temperature in the dog ear canal at resting conditions varies from 37 to 40 °C^46^. To make the results comparable with results achieved for different microbial cultures in different experiments, the temperature 37 °C was chosen.

The initial suspension was prepared in phosphate buffered saline (PBS) with a concentration of 1 McFarland (3∙10^8^ CFU/ml). From the initial suspension, 50 µl was transferred into a test tube with 4950 µl of the tested PTL, standard solution of reactive species or PTL alternative (artAC_air_, artDC). At this moment, the stopwatch was turned on. The sampling was carried out at selected contact times 10, 30, 60, 120, 180, 240 min and 24 h. At each time point 500 µl of the sample was transferred into 4500 µl of PBS. Ten-fold dilutions (10 ^[– [1^ to 10 ^[– [4^) were prepared and 100 µl of the diluted culture was subsequently inoculated onto the agar plate and spread over the entire surface. Control samples were prepared in a similar manner, but instead of inoculation into the PTL (or other testing solution), the 50 µl of the initial suspension was inoculated into the PBS. The sampling of controls was carried out at selected contact times 10 min and 24 h. Control of optimal cell growth (growth curve) was also prepared, where the initial suspension was inoculated into a corresponding liquid nutrient medium. The sampling was carried out at selected contact times of 2, 4, 8 and 24 h. Dilution row was carried out by decimal dilution up to 10^− 6^. All samples were subsequently cultivated at 37 °C for 24 h. The plate count method, using the software Aurora^47^ from HexTech Research s.r.o., was chosen to determine antimicrobial efficiency. All selected microorganisms were tested in this way. The measurements with each of the testing solutions were done for at least 3 biological replicates (composed of 3 technical replicates).

## Results and discussion

### Composition of the PTL depends on the plasma treatment

As mentioned in the introduction, the composition of the plasma treated liquids depends on the plasma system used for their preparation (see Table 3). The plasma systems generating plasma inside the liquid (DC, AC or AC_air_) and the dielectric barrier discharge with the liquid electrode (PTL_DBD_) were compared in terms of the reactive species, conductivity, and pH. The DC plasma system provided a higher concentration of hydrogen peroxide compared to the other systems. This can be associated with a high production of hydroxyl radicals coming from direct ionization of water molecules, and their subsequent mutual reaction forming hydrogen peroxide. On the other hand, the DBD system provided PTL with an increased amount of nitrogen species (both nitrites and nitrates) via the gas phase chemistry of plasma in air above the liquid surface. Even with an increased concentration of RONS, the pH of both PTLs remained relatively high reaching approximately the value of 5.3 (see Table 3). The AC plasma inside water system was chosen because of its lower impact on electrode degradation and improved operation safety. However, such PTL contained relatively low concentrations of RONS except for hydrogen peroxide, whose concentration was still higher than in case of PTL_DBD_, but simultaneously much lower than in PTL_DC_. Therefore, the modification of the AC plasma system with the dry artificial air bubbling (oxygen and nitrogen in the ratio of 1:4) into the discharge region was employed. This AC_air_ plasma system provided both significantly higher concentrations of nitrogen species (especially nitrites) while maintaining the high concentration of hydrogen peroxide. Moreover, the produced combination of reactive species substantially decreased the pH value of the treated liquid from 5.8 to 3.5. This increased acidity might induce formation of peroxynitric acid or peroxynitrites, which are important species for decontamination processes^66^. Generally, the plasma treatment of the physiological solution decreased the pH value and increased conductivity, with the most impact observed for the AC_air_ plasma system. This is in contrast with the treatment of tap water, especially mineral-rich water, which has a buffering capacity. As reported by Čechová et al.^21^ in a recent study, treatment of mineral-rich tap water may result in relatively stable pH values or even a slight increase.

**Table 3:** Composition of non-treated physiological solution (PS), PTL_DC_, PTL_AC_, PTL_ACair_, and PTL_DBD_ (LOD = limit of detection).

	PS	PTL_DC_	PTL_AC_	PTL_ACair_	PTL_DBD_
*σ* [mS∙cm^− 1^]	14.9 ± 0.3	16.7 ± 0.5	16.0 ± 0.3	16.5 ± 0.3	15.3 ± 0.2
pH [-]	6.0	5.2 ± 0.3	5.8 ± 0.2	3.5 ± 0.2	5.3 ± 0,3
H_2_O_2_ [mg∙l^− 1^]		16.0 ± 3.0	2.9 ± 0.3	1.7 ± 0.3	0.7 ± 0.2
NO_2_^−^ [mg∙l^− 1^]		0.2 ± 0.2	0.03 ± 0.01	11.0 ± 3.0	1.3 ± 0.5
NO_3_^−^ [mg∙l^− 1^]		25.0 ± 7.0	≤LOD	42.0 ± 3.0	52.0 ± 6.0

### Selection of effective PTL

When testing the antimicrobial efficacy of PTLs prepared by different plasma sources (DC, AC, and DBD), the contact times: 10, 30, 60, 120, 180 and 240 min and clinical isolates of *P. aeruginosa* and *S. pseudintermedius* were used.

**Fig. 2:**
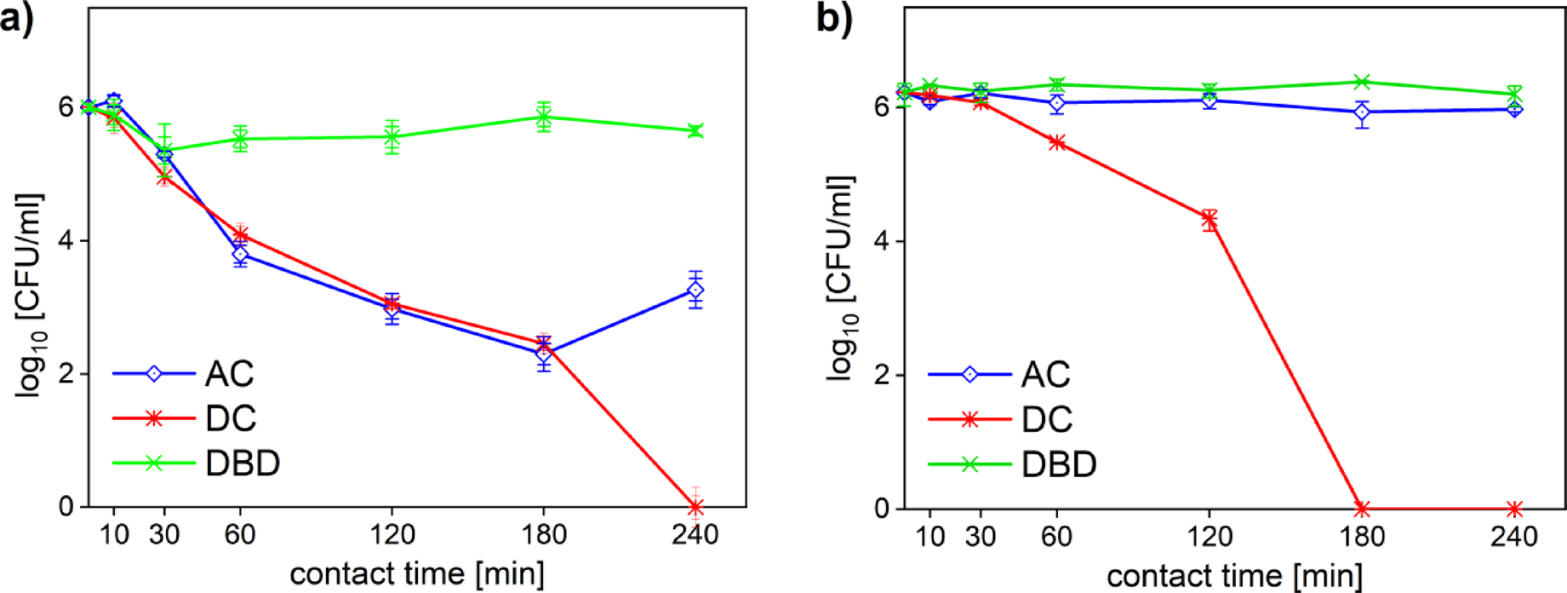
Results of decontamination efficacy of tested PTLs shown for clinal isolates of (**a**) *P. aeruginosa* and (**b**) *S. pseudintermedius*.

The solution treated with the pinhole discharge with DC high voltage source (PTL_DC_) was found to be the most effective. For the gram-negative bacteria *P. aeruginosa* in contact with PTL_DC_, continuous decrease in cell viability with increased exposure time was observed. Compared to the other tested PTLs, where only 2–3 log_10_ reduction was observed even after 4 h of exposure (see Fig. 2). For the gram-positive bacteria *S. pseudintermedius*, a 6 log_10_ reduction was achieved after 3 h, whereas the other PTLs showed no significant effect (see Fig. 2).

After the first set of experiments done on clinical isolates, the PTL_DC_ and the PTL_AC_ were selected for further measurements. However, the PTL_AC_ needed to be improved, to provide more inhibition efficacy. As can be seen on the Fig. 2a, a slight increase in colony count was observed. This could have been caused by the adaptation of the microorganism to the environment of the PTL_AC_ (pH 5.8) or depletion of the RONS. Therefore, the air bubbling was added to the plasma system to produce PTL_ACair_ containing more RONS and increased acidity.

The following data show the results of experiments performed with the reference strains *P. aeruginosa* (ATCC 27853) and *S. aureus* (ATCC 29213). More measurements were done with the reference strains to make the data comparable with other studies.

### Efficacy of the PTL compared to the standard solutions of RONS

The antimicrobial efficacy of plasma-treated liquids (PTLs) is driven by a complex mixture of reactive oxygen and nitrogen species (RONS), each affecting key microbial functions. Hydrogen peroxide (H_2_O_2_), one of the dominant long-lived species in PTLs, readily diffuses across cell membranes and induces oxidative stress by damaging DNA, proteins, and lipids. Nitrites (NO_2_⁻) and nitrates (NO_3_⁻), as representatives of reactive nitrogen species (RNS), affect pH and redox balance through nitration and oxidative inactivation of critical biomolecules (mainly redox-sensitive enzymes)^12,15^. Therefore, their impact on the bacterial strains was studied to help determine the major RONS involved in the antimicrobial effects of PTL.

### *Pseudomonas aeruginosa*

**Fig. 3:**
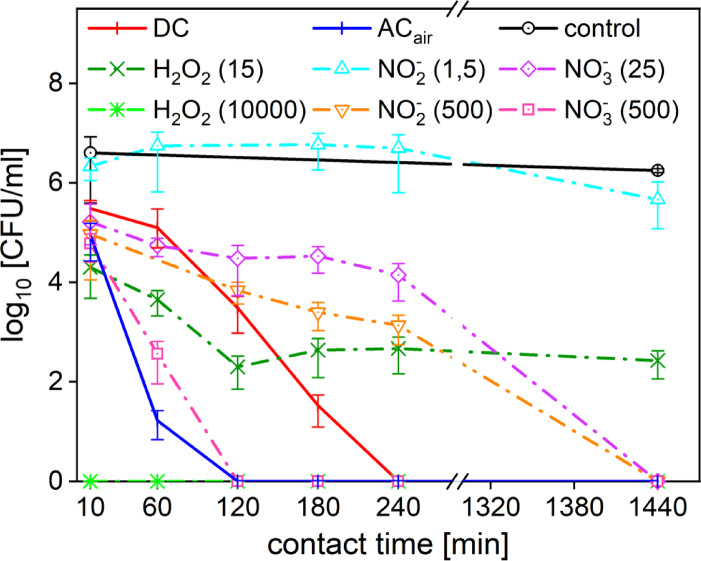
Reduction of *P. aeruginosa* viability upon exposure to PTL_DC_ (DC), PTL_ACair_ (AC_air_), standard solutions of hydrogen peroxide, nitrites, nitrates, and control measurement inoculated in PBS (control). The standards of individual reactive species with concentrations comparable to the ones in PTLs are listed in the middle row, and the highest tested concentration in the bottom row.

The effects of PTL_DC_ and the standard solutions of hydrogen peroxide, nitrites, nitrates at different concentrations on *Pseudomonas aeruginosa* are shown in Fig. 3. Complete inhibition (6log_10_ reduction) with the use of PTL_DC_ was achieved after 4 h of contact. In comparison to the individual reactive species standards (concentrations corresponding approximately to those in PTL_DC_), the PTL_DC_ had significantly better effects. The improvement of the PTL_AC_ to PTL_ACair_ exhibited increased antimicrobial efficacy, even when compared to PTL_DC_. A complete inhibition of microbial growth was achieved after 2 h (see Fig. 3).

According to the testing with standards of reactive species, *Pseudomonas aeruginosa* seemed very susceptible to both hydrogen peroxide and nitrates, which may help untangle the important difference in the effects of PTLs. A high inhibition efficacy of the hydrogen peroxide was observed even for very small concentration. Concentration of 15 mg∙l^− 1^ H_2_O_2_ (corresponding to the concentration in PTL_DC_) resulted in 3log_10_ reduction after 2 h, which then remained almost unchanged. For the intermediate concentrations (100 and 1000 mg∙l^− 1^ of H_2_O_2_) continual decline of colonies and subsequent complete reduction (6log_10_) with no recovery was observed after 2 h. The highest tested concentration 10,000 mg∙l^− 1^ led to a 6log_10_ reduction within just 10 min, and no bacterial recovery was observed. Considering the effects of RNS solutions, nitrates were generally more efficient than nitrites. Nitrites at 1.5 mg∙l^− 1^ had little to no effect, while 500 mg∙l^− 1^ caused significant inhibition, but only after 24 h. In contrast, nitrates at a concentration of 500 mg∙l^− 1^ caused complete bacterial reduction within 2 h. Even at very low nitrate concentration of 25 mg∙l^− 1^, a complete inhibition was achieved after 24 h. This means that a 25 mg∙l^− 1^ nitrate solution showed a comparable effect to a 500 mg∙l^− 1^ nitrite solution. Thus, *P. aeruginosa* may be expected to be more susceptible to nitrates even at low concentrations. For the full spectrum of different tested concentrations of H_2_O_2_, NO_2_⁻ and NO_3_⁻, see Fig-9 in the Appendix.

*P. aeruginosa* exhibited greater sensitivity to all the tested RONS solutions. However, besides the highest concentrations of the hydrogen peroxide (10000 mg∙l^− 1^) and nitrates (500 mg∙l^− 1^), the PTL_ACair_ was the most efficient with the 6 log_10_ reduction in 2 h of contact.

The difference in the composition and effects of the two PTLs is an outcome of the different plasma treatment type. There is twice as much nitrate in PTL_ACair_ compared to PTL_DC_ but ten times less hydrogen peroxide (see Table 1). Additionally, PTL_ACair_ contains two orders of magnitude higher concentration of nitrites and has a lower pH. This made us assume that not only the high concentration of nitrates and hydrogen peroxide (as in the PTL_DC_) was important for the decontamination. Also, the presence of high concentration of nitrites together with the lower pH provided a highly reactive environment for different chemical reactions. In previous studies it was found that these conditions facilitate peroxynitrite formation^14^. Peroxynitrite is well known for its highly reactive nature. It nitrates tyrosines, oxidizes DNA and enzymes, contributing to widespread disruption of cellular processes, including membrane integrity, energy metabolism, and DNA replication^15^.

Further support for our findings comes from the study by Shaw et al.^48^who demonstrated that the co-presence of nitrites (NO_2_⁻) and hydrogen peroxide (H_2_O_2_) in plasma-treated water leads to the formation of peroxynitric acid (O₂NOOH), which subsequently decomposes into superoxide anion (O_2_⁻). Shaw et al. investigated the antimicrobial effects of plasma-treated water (PTW) enriched with reactive nitrogen species (RNS) against *Escherichia coli.* They showed that PTW with high levels of both NO_2_⁻ and H_2_O_2_ exhibited significantly greater antimicrobial activity than formulations enriched in H_2_O_2_ alone, despite similar pH levels. Notably, they reported that at pH ~ 4.5 (comparable to that of our PTLs) the half-life of O_2_NOOH is approximately 1.6 min^14^suggesting that it can persist long enough to contribute meaningfully to bacterial inhibition via O_2_⁻ formation. This aligns with our observation that *P. aeruginosa* was more susceptible to nitrite-rich PTL_ACair_, supporting the idea that secondary reaction products such as O_2_NOOH and O_2_⁻ may play a critical role in the enhanced efficacy of these formulations. While we did not directly measure these intermediates, the inability of single-component solutions to reproduce the full antimicrobial effect of PTLs points toward similar synergistic mechanisms in our system.

Thus, it may be both the higher concentration of nitrates and nitrites with some essential concentration of hydrogen peroxide at lower pH that leads to better efficacy of PTL_ACair_. Altogether making the PTL_ACair_ a unique and promising agent for decontamination.

### *Staphylococcus aureus*

**Fig. 4:**
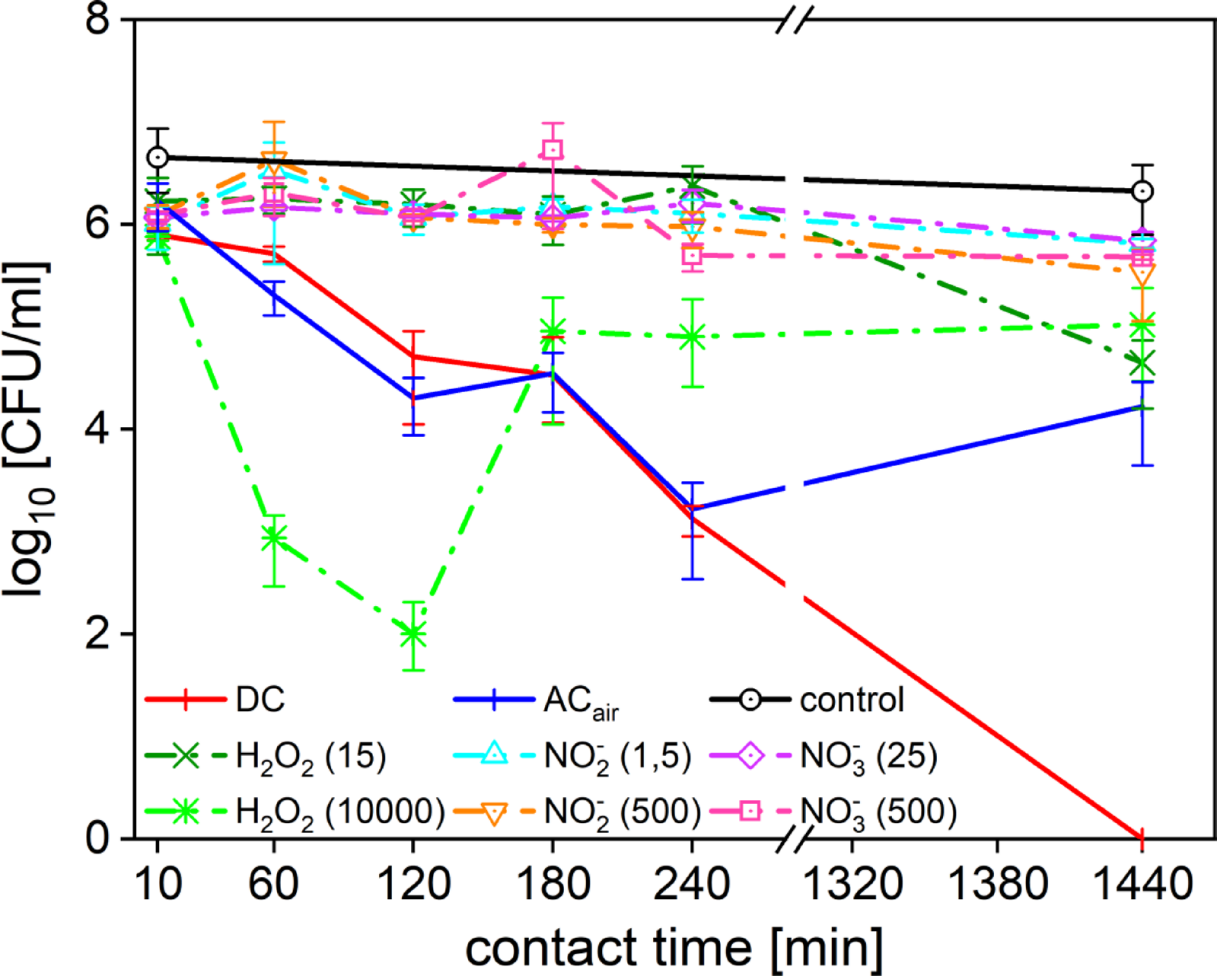
Reduction of S. aureus viability upon exposure to PTL_DC_ (DC), PTL_ACair_ (AC_air_), standard solutions of hydrogen peroxide, nitrites, nitrates, and control measurement inoculated in PBS (control). The standards of individual reactive species with concentrations comparable to the ones in PTLs are listed in the middle row, and the highest tested concentration in the bottom row.

Testing the effects of PTLs and different concentrations of standard solutions (nitrites, nitrates and hydrogen peroxide) on *S. aures* showed that PTL_DC_ was the most effective during the long‑term exposure (see Fig. 4). A gradual decrease of culturable cells resulted in a 3 log_10_ reduction in 4 h of contact time with PTL_DC_. After 24 h of contact, a complete inhibition (more than 6 log_10_ reduction) was observed. Therefore, it can be concluded that after 24 h of exposure to PTL_DC_, bacteria were completely inhibited. Compared to the data collected for the clinical isolate of *S. pseudintermedius*, where the PTL_DC_ exhibited better efficacy and a 6 log_10_ decontamination was observed after 3 h of exposure. This can be attributed to the higher susceptibility of the clinical isolate or to the strain specificity and increased resistance of the reference strain.

Regarding the PTL_ACair_, the largest decrease in colony count occurred at 4 h by less than 3 log_10_, similarly to PTL_DC_. However, unlike PTL_DC_, PTL_ACair_ did not show such good effects after the prolonged exposure. A slight increase in the number of colonies was observed after the 24 h exposure. A similar behaviour was observed for *P. aeruginosa* (the clinical isolate) shown in Fig. 2a. The increased number of *S. aureus* colonies after the 24 h exposure may be caused by different adaptation mechanisms of the bacteria to their environment. The PTL_ACair_ is more acidic than the PTL_DC_. However, it has been proposed in different studies^49–51^ that *S. aureus* can adapt to lower pH and exhibit unimpaired viability also in slightly acidic environments. This factor has also been tested and the results will be discussed later in the text.

Differences between the two PTLs and their antimicrobial efficacies may derive from their distinct preparation methods, which influence chemical composition, pH, and conductivity. Notably in case of *S. aureus*, the key difference in relation to these observations may lie in the concentration of hydrogen peroxide.

To study this phenomenon, the influence of individual particles and their effects were taken into consideration. For *Staphylococcus aureus*, nitrate and nitrite solutions did not lead to any significant reduction in colony counts even at the highest tested concentration of 500 mg∙l^− 1^ (see Fig. 4). A standard hydrogen peroxide solution at 15 mg∙l^− 1^ (corresponding to the concentration found in PTL_DC_) showed no inhibitory effect; on the contrary, a slight stimulatory effect could be observed. Also for other intermediate concentrations (25‒100 mg∙l^− 1^), an increase in viability was observed over longer exposures, which may be explained by a hormetic effect. This means that RONS can have stimulatory and inhibitory impact depending on the concentration; meaning that low levels of RONS may induce a beneficial stress response and promote cell growth. Thus, we assume that the lower concentrations of H_2_O_2_ (up to 100 mg∙l^− 1^) may cause the stimulative stress response, and increased cell replication observed as increased number of colonies, e.g., for 50 or 100 mg∙l^− 1^ at 4 h of the exposure. This trend has also been observed and investigated by Chang et al. in their recent study^52^. At a higher concentration of 10,000 mg∙l^− 1^ (1% w/v), hydrogen peroxide induced a 4 log_10_ reduction in viable cells after 2 h. However, with longer exposure times, colony counts began to rise and then stabilized at values lower than the control. Our results suggest that *S. aureus* exhibits a high degree of adaptability to oxidative stress, and even at 10,000 mg∙l^− 1^, complete inhibition was not sustained over time, suggesting that its MIC for hydrogen peroxide may be higher than previously found in literature^39^. As such, disinfectant solutions typically use H_2_O_2_ at concentrations of 3% o more^53^which were beyond the scope of this study.

The partial recovery studied for the solutions of H_2_O_2_ is likely due to the bacterium’s adaptive response, including the upregulation of the production and activity of the enzyme catalase^52,54^. Catalase is expressed by the cell as a defence mechanism against oxidative stress by the decomposition of hydrogen peroxide into water and oxygen^55^. The upregulation of this metabolic pathway because of the CAP or PTL treatment has also been observed in several different studies^56,57^. Thus, we assume that a similar mechanism may be occurring in the PTLs tested in this study, potentially contributing to the increase colony count observed for PTL_ACair_ at 24 h. The concentration of hydrogen peroxide is much lower in PTL_ACair_ than in PTL_DC_ (see Table 1). Then, the lower concentration of H_2_O_2_ may be overcome by the bacterial adaptation mechanisms (as shown for the standard solutions of H_2_O_2_) and lead to bacterial revitalization. Higher concentration of hydrogen peroxide in the presence of other RONS can lead to formation of different secondary reactive species (including peroxynitrites, superoxide, etc.), that have been shown to have strong antimicrobial properties^10^. Thus, the presence of higher hydrogen peroxide concentration in cooperation with other factors for an extended time period (such as 24 h), can trigger too much stress and cause the cell death, which could be the reason why *Staphylococcus aureus* was more sensitive to PTL_DC_.

In summary, the effects of PTLs have been shown to be more permanent and effective compared to the individual reactive species. This may be attributed to the complex composition and content of various reactive species produced in plasma chemical reactions among different reactive species at specific conditions.

### Efficacy of the PTLs compared to the solutions simulating the chemical composition of the tested PTL

#### Pseudomonas aeruginosa

**Fig. 5:**
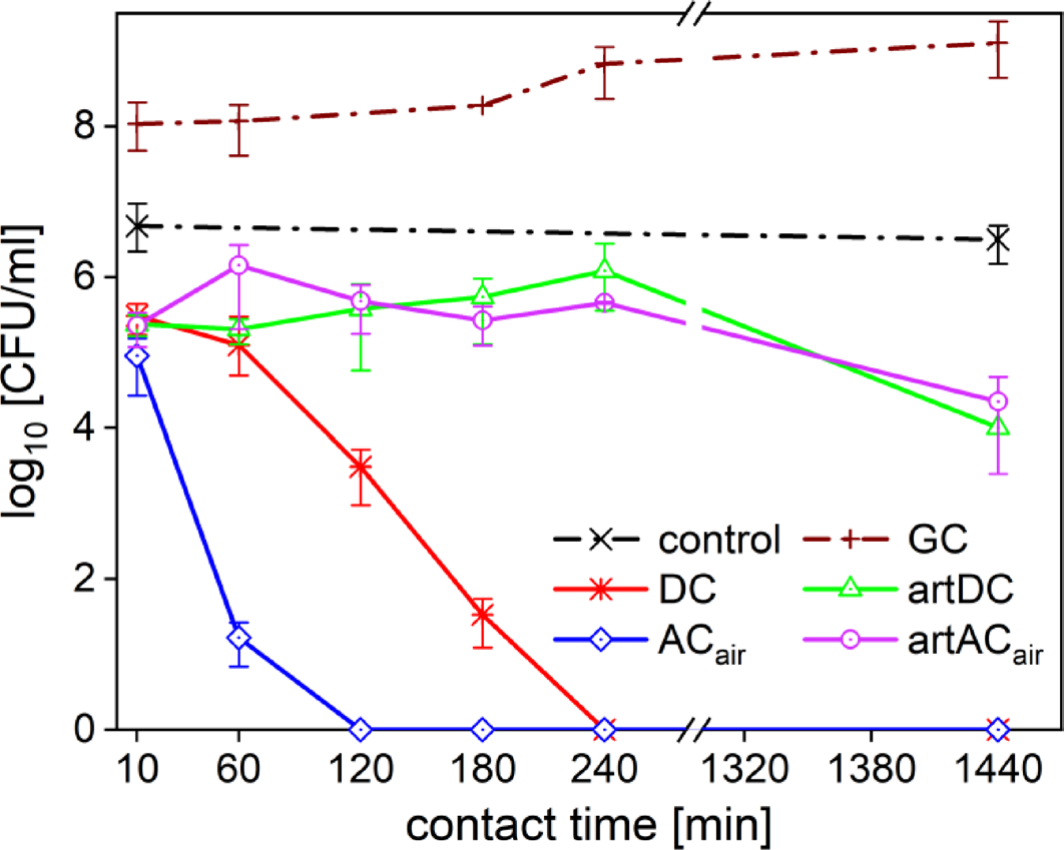
Comparison of the inhibition efficacy of plasma treated liquids PTL_DC_ (DC), PTL_ACair_ (AC_air_) and solutions simulating their composition (artDC, and artAC_air_) against *P. aeruginosa*. Control measurement inoculated in PBS (control) and control inoculated in nutrient medium (GC).

The effects of solutions simulating the composition of the most efficient PTLs prepared in two inside liquid plasma systems (DC and AC_air_) were also tested. The observed effects of solutions simulating the chemical composition of PTL (artDC and artAC_air_) on *P. aeruginosa* are shown together with the effects of the studied PTLs (see Fig. 5). It was found that the artificial alternatives artDC and artAC_air_ did not have as good antimicrobial effects on *P. aeruginosa* as the real PTLs. Both simulating solutions showed similar effects with the greatest decrease in colony count (less than 2 log_10_) at the 24 h exposure.

To compare the antimicrobial efficacy of PTLs to the cells living in their favourable environment, the growth control (the growth curve, GC) was included. In this case the control was inoculated in the nutrient medium, where the bacteria could thrive and replicate (as in the real case scenario).

The largest difference in colony counts between PTL_DC_ and its artificial alternative (artDC) occurred in 4 h, where full decontamination was achieved using the PTL_DC_. When compared to the bacterial growth in optimal conditions (the growth curve), an 8.5 log_10_ reduction would be assigned to the 4 h exposure. Comparing the effects of PTL_ACair_ and artAC_air_, the largest difference in the number of colonies was noticed after 2 h of the exposure. A difference of 6 log_10_ (a complete inhibition) and of 8 log_10_ compared the control was observed in the nutrient medium. For both PTLs, the difference between the bacterial viability in the PTL and the nutrient medium (GC) was more than 9 log_10_ for the 24 h exposure. For both solutions simulating the chemical composition of PTL (artPTLs) compared to the control inoculated in the nutrient medium (GC), a 5 log_10_ reduction after 24 h was recorded.

#### Staphylococcus aureus

**Fig. 6:**
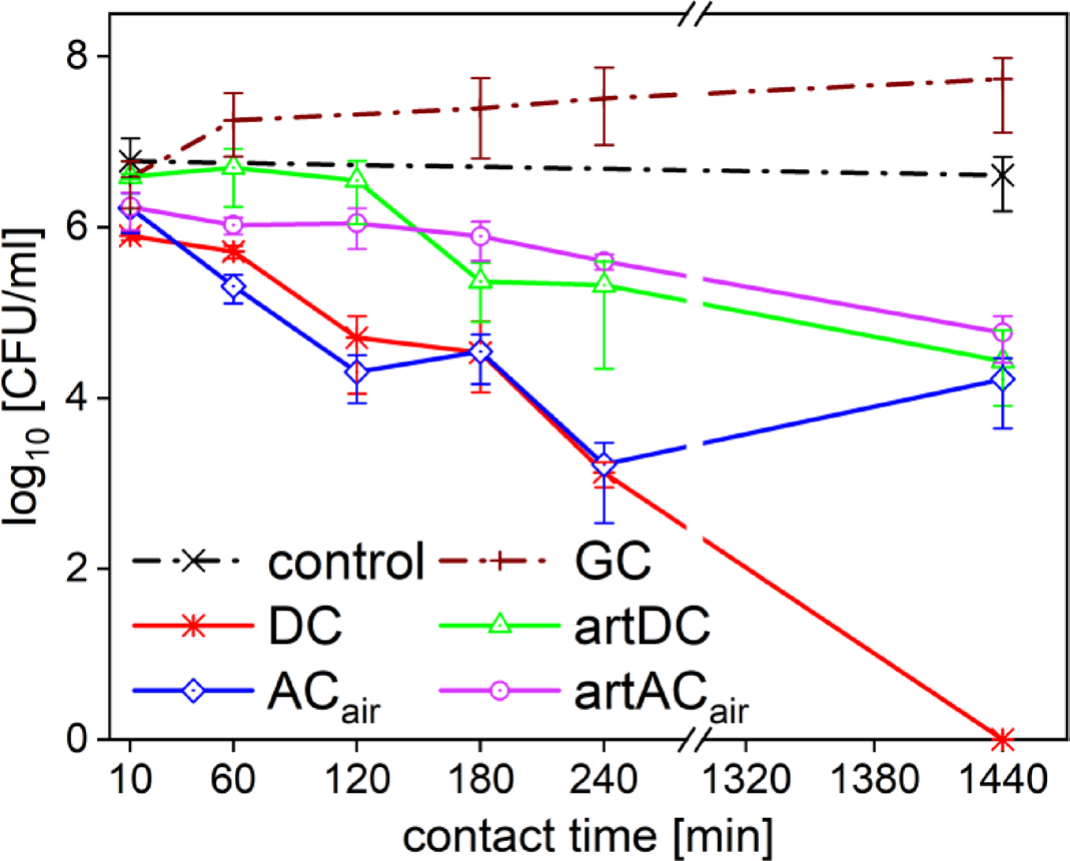
Comparison of inhibition efficacy of the plasma treated liquids PTL_DC_ (DC), PTL_ACair_ (AC_air_) and solutions simulating their composition (artAC_air_, and artDC) against S. aureus. Control measurement inoculated in PBS (control) and control inoculated in nutrient medium (GC) shown in dash-dotted line.

As shown above for *P. aeruginosa*, the artificially created solutions did not show as good antimicrobial activity as PTLs themselves neither for *S. aureus*. The decrease in the number of colonies was slower in artDC and artAC_air_ reaching the maximum decrease of 2 log_10_ after 24 h compared to the control measurement (Fig. 6). The difference between PTL_DC_ and artDC at the 24 h contact time was about 4.5 log_10_. The greatest difference in the antimicrobial activity (2 log_10_) for PTL_ACair_ and artAC_air_ was achieved in 4 h of the exposure. However, the number of countable colonies was comparable after 24 h.

To compare the antimicrobial efficacy of PTLs with the cells in their favourable environment, the growth control (the growth curve, GC) was included. In this case, the control was inoculated in the nutrient medium, where the bacteria could thrive and replicate (as in a real case scenario). Comparing the efficacy of the PTLs and the GC control after 24 h, PTL_DC_ achieved a difference of almost 8 log_10_, while PTL_ACair_ and artificial alternatives 3.5 log_10_, only.

The antimicrobial efficacy of plasma-treated liquids (PTLs) is driven by a complex interplay of reactive oxygen and nitrogen species (RONS), each with distinct effects on bacterial cell components. Considering the results achieved, we can conclude that the prepared plasma treated liquids show better antimicrobial activity than their chemically prepared alternatives. The findings of this study demonstrate that prepared PTLs contain certain attributes that artificially created solutions do not have. This may be due to the presence of other particles formed during the treatment of liquids (e.g., hydrogen ions, peroxynitrites, superoxide, etc.) which, among other things, also lower the pH value (see Table 3). Therefore, we conclude that the composition of the plasma treated liquids is unique and cannot be replaced by a mere mixing of the major compounds detected in the PTL.

### Efficacy of the PTLs compared to the solutions simulating different pH values

**Fig. 7:**
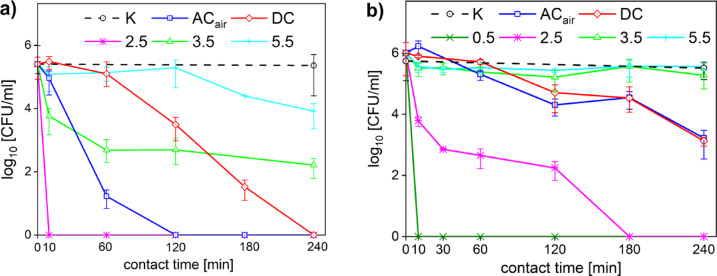
Inhibition efficacy of pH adjusted solutions (from 0.5 to 5.5) for (**a**) P. aueruginosa and (**b**) S. aureus compared to the inhibition efficacy of the PTL_ACair_ (AC_air_) and PTL_DC_ (DC).

The environmental pH levels significantly impact bacterial survival, growth and overall vitality by influencing cellular processes and the effects of oxidative stress. Bacteria respond to pH changes by regulating gene expression and protein profiles^18^. Thus, the acidic pH of PTLs is another factor contributing to their antimicrobial efficacy. To further study the different synergistic agents of PTL, the effect of different pH values was investigated. In this study, pH values of tested PTLs were 3.5 (PTL_ACair_) and 5.2 (PTL_DC_). As can be seen in Fig. 7b for *S. aureus*, its viability was unaffected at pH levels above 3. The pH values (pH = 3.5 and pH = 5.5) comparable to selected PTL_ACair_ and PTL_DC_ did not exhibit any inhibition efficacy. However, a significant decrease in cell viability was observed for the solutions with pH lower than 2.5. These findings are in alignment with previous studies which have shown that *S. aureus* demonstrates notable acid tolerance, supported by an integrated acid response network. This includes membrane remodelling, proton efflux mechanisms, and various acid-neutralizing metabolic pathways. These mechanisms help to maintain cytoplasmic pH and protect cellular integrity under acidic stress, enabling survival in transiently low pH environments such as inflamed tissue or infected wounds^18,58^. However, at lower pH and concurrent oxidative stress, membrane integrity and metabolic activity can be severely compromised as shown for other microbial species^16,48^. The synergistic effects of RONS and acidic environment have been shown in recent findings by Xie et al.^17^. The study confirmed that synergistic interactions between acidic stress and both short and long-lived RONS led to substantial membrane depolarization and protein oxidation in *Staphylococcus simulans*, species closely related to *S. aureus*. Acidic conditions alone induced transient membrane stress, but in combination with RONS, caused significant, long-lasting impairment of membrane function. This is in line with our observation that *S. aureus* was more sensitive to PTL_DC_ with higher concentration of hydrogen peroxide and lower pH conditions that likely overwhelmed its catalase-based ROS defences and induced oxidative membrane damage.

In contrast, *Pseudomonas aeruginosa* is more sensitive to acidic environments, with noticeable viability loss at pH values below 5. For *P. aeruginosa* (Fig. 7a) the pH = 5.5 did not alter the cell viability but the solutions with the lower pH exhibited a strong inhibition efficacy. This aligns with previous findings showing that *P. aeruginosa* has limited ability to maintain pH homeostasis under acidic stress, which can impair cellular function and increase susceptibility to additional stressors such as reactive nitrogen species^44,59^. This susceptibility to acidic environment may also be the reason why the PTL_ACair_ was more effecient in the inhibition of *P. aeruginosa*, compared to PTL_DC_. The PTL_ACair_ (pH = 3.5) also exhibited stronger antimicrobial efficacy than the testing solution with pH = 3.5. This can be attributed to the synergistic effects of pH and the reactive species in the PTL as discussed above. Therefore, although acidity alone did not account for the full antimicrobial effect, it may contribute to the enhanced susceptibility of *P. aeruginosa* to PTLs with lower pH. These results support the idea that pH plays a significant role in microbial inhibition of certain microbial species, but it is not solely responsible for the antimicrobial effects of the PTL.

### Comparison of the Inhibition efficacy for *P. aeruginosa* and *S. aureus*

The gram-negative *P. aeruginosa* was observed to be more susceptible to all treated solutions, including PTLs, standard solutions and the solutions simulating the PTL composition.

When speaking about the standard solutions, the bacterium *P. aeruginosa* was more sensitive to the standard solutions of nitrates, nitrites and mainly hydrogen peroxide, compared to *S. aureus*, where none of these solutions showed any significant antimicrobial activity.

When comparing the PTLs in their efficacy for each bacterium, the PTL_DC_ works better than PTL_ACair_ for *S. aureus* and vice versa PTL_ACair_ works better than PTL_DC_ for *P. aeruginosa*. The difference in the effects of the two PTLs is most probably due to the different modes of preparation resulting in different compositions of the two tested PTLs. The PTL_DC_ is more hydrogen peroxide rich with higher pH (approx. 5.5). Whereas the PTL_ACair_ is more abundant in nitrates and nitrites and has lower pH (approx. 3.5). There are twice as many nitrates in PTL_ACair_ compared to PTL_DC_ (see Table 1). The situation is the opposite for hydrogen peroxide and an order of magnitude less hydrogen peroxide is produced in PTL_ACair_. Additionally, concentration of nitrites is two orders higher in PTL_ACair_ than in PTL_DC_.

From the results of standard solutions of reactive species, *P. aeruginosa* is susceptible to both hydrogen peroxide and nitrates, which may be the decisive difference in the effects of PTLs themselves. Higher concentration of nitrates and nitrites, especially, in combination with lower pH may be the reason for the better effect of PTL_ACair_ compared to PTL_DC_. When considering *S. aureus*, the only microbial load reduction with the use of standard solutions of the reactive species was observed for very high amounts of hydrogen peroxide. Since the PTL_DC_ contains more hydrogen peroxide than PTL_ACair_, it may result in better inhibition efficacy. The role of acidity of the environment may also play an essential role, where *S. aureus* can adapt to different pH conditions, as discussed in the paragraph above.

**Fig. 8:**
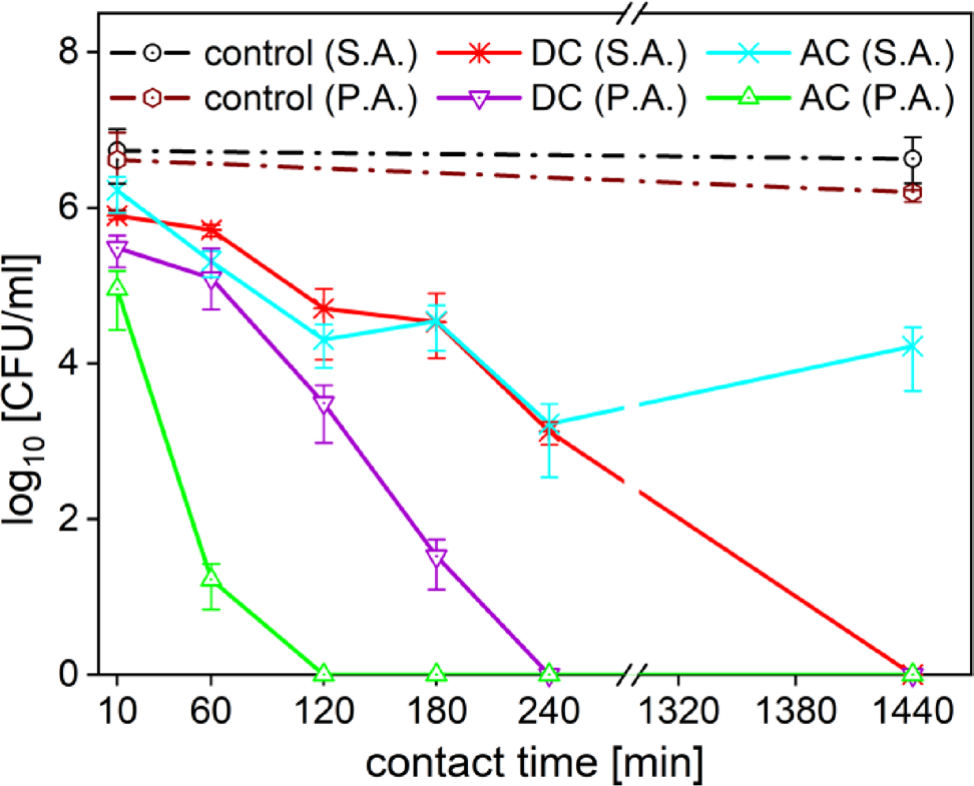
Comparison of the inhibition efficacy for the two different bacteria *S. aureus* and *P. aeruginosa*, where PTL_DC_ (DC S.A.), PTL_ACair_ (AC S.A.) and control (control S.A.) refer to *S. aureus* and PTL_DC_ (DC P.A.), PTL_ACair_ (AC P.A.) and control (control P.A.) represent the results for *P. aeruginosa*.

**Fig. 9:**
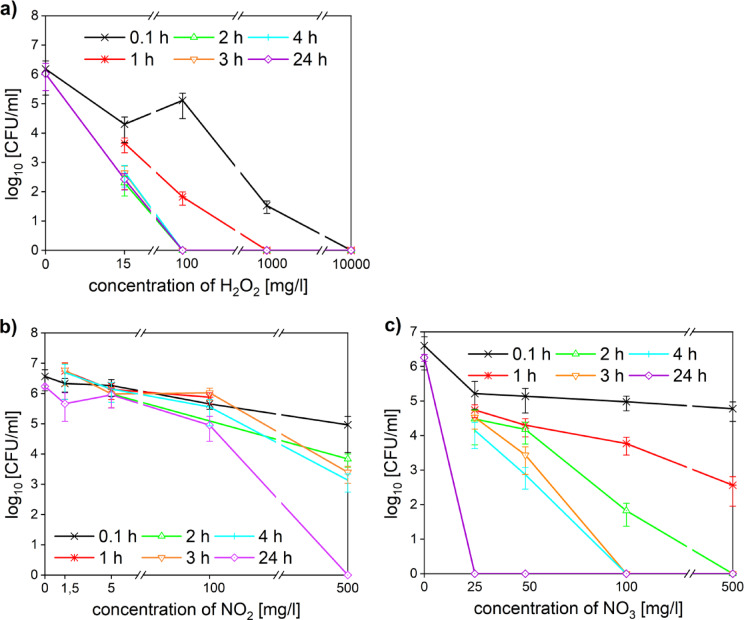
Reduction in *P. aeruginosa* viability using the standard solutions of selected RONS in range of concentrations shown for different exposure times (**a**) hydrogen peroxide (H_2_O_2_,) (**b**) nitrites (NO_2_,) (**c**) nitrates (NO_3_).

Comparing the PTLs in general antimicrobial efficacy against *P. aeruginosa* and *S. aureus*, it can be said that PTL_DC_ exhibits better inhibition efficacy for *P. aeruginosa* (see Fig. 8). While for *S. aureus* each hour of exposure to PTL_DC_ reduced the number of colonies by 1 log_10_, for *P. aeruginosa* the inhibition effect was twofold. Complete inhibition of *P. aeruginosa* was achieved after 4 h of the exposure, compared to 24 h for *S. aureus*. Similarly, PTL_ACair_ had significantly better antimicrobial activity against *P. aeruginosa* than against *S. aureus*. When PTL_ACair_ was in contact with *S. aureus*, the largest decrease of 3 log_10_ was recorded at 4 h, while *P. aeruginosa* was completely inhibited after 2 h of the exposure, only. Since both bacteria have a different structure and composition of the cell wall and distinct antioxidant defence mechanisms (which influence their ability to cope with oxidative stress), there is also a difference in the effects of RONS solutions and PTL during the exposure.

To correlate the results observed for different PTLs or other studied solutions, it’s important to study how the different microbial species cope with oxidative stress. The antioxidant capacities of the studied bacteria *Staphylococcus aureus* and *Pseudomonas aeruginosa* exhibit distinct oxidative stress defence mechanisms, which may help to explain their differing susceptibilities to plasma-treated liquids (PTLs). *S. aureus* relies primarily on catalase activity and the membrane-bound antioxidant pigment staphyloxanthin to protect against reactive oxygen species (ROS), particularly hydrogen peroxide and singlet oxygen. Its oxidative stress response is governed by regulators such as PerR and SarA, and it possesses a relatively limited glutathione system compared to gram-negative bacteria^52,60^. Our findings indicate that *S. aureus* was more susceptible to PTLs containing elevated concentrations of hydrogen peroxide, which aligns with its reliance on catalase as a primary defence mechanism and relatively limited intracellular redox buffering capacity^52^. Once catalase is overwhelmed or inactivated, *S. aureus* becomes vulnerable to oxidative damage, particularly from hydrogen peroxide and hydroxyl radicals.

In contrast, *P. aeruginosa* employs a more robust and multifaceted defence, including multiple catalase isoenzymes (e.g., KatA, KatB), high superoxide dismutase activity, and an active glutathione-based redox system. It also benefits from global regulators such as OxyR and SoxR/S, which tightly coordinate responses to oxidative and nitrosative stress^61^. These capabilities, along with biofilm formation and efflux mechanisms, contribute to its general resilience against oxidative damage^62^. Despite its strong ROS detoxification systems, *P. aeruginosa* may be more vulnerable to nitrosative stress, which can impair essential cellular functions by modifying iron-sulphur clusters, thiols, or regulatory proteins^63^. Thus, *P. aeruginosa* appears to be more susceptible to RNS-mediated stress and in combination with sensitivity to low pH, it exhibited higher susceptibility to PTL_ACair_ rich in RNS and low pH.

Interestingly, despite *P. aeruginosa* possessed more robust antioxidant defences, it was more susceptible to PTL and other RONS solutions than *S. aureus*. This suggests that other factors such as the outer membrane composition and permeability may play a more critical role in initial inactivation. Since the cell envelope represents the first line of defence against external stressors, its structure and chemical properties could significantly influence how RONS interact with and penetrate bacterial cells. Although the present study cannot conclusively determine whether antioxidant capacity or the cell wall composition is the dominant factor, our findings align with previous observations that gram-negative bacteria (*P. aeruginosa*) tend to be more sensitive to the plasma treatment (PTL and CAP) than gram-positive bacteria (*S. aureus*). This highlights the complex interplay of structural and biochemical defences in determining bacterial resilience. These findings underscore the importance of PTL composition in determining antimicrobial efficacy and suggest that tailoring RONS profiles to exploit specific weaknesses in bacterial antioxidant systems could enhance the efficacy of plasma-based treatments.

## Conclusion

The antimicrobial efficacy of plasma-treated liquids (PTLs) is driven by a complex interplay of reactive oxygen and nitrogen species (RONS), each with distinct effects on bacterial cell components. These effects are significantly intensified under acidic conditions, which promote the formation and stability of certain RONS, lower bacterial intracellular pH homeostasis, and increased membrane vulnerability ^15 16^. To study the role of different aspects of PTLs (RONS and pH) in decontamination efficacy, this study compared the different PTLs with various simulating solutions.

Plasma treated liquids were prepared from sterile saline solution using plasma systems with different plasma-liquid interaction (plasma above or inside the treated liquid). The concentration of nitrates, nitrites, hydrogen peroxide, conductivity and the pH value were determined. The most efficient plasma treated liquids (PTL_DC_ and PTL_AC_ prepared by the inside liquid plasma systems) were selected from the preliminary measurements with clinical isolates of *Staphylococcus pseudintermedius* and *Pseudomonas aeruginosa*. The PTL_AC_ was further modified to PTL_ACair_ by adding air bubbling into the plasma system to render better antimicrobial efficacy.

Subsequently, these PTLs were tested with reference strains *Staphylococcus aureus* (ATCC 29213) and *Pseudomonas aeruginosa* (ATCC 27853). Standard solutions of nitrates, nitrites, and hydrogen peroxide were prepared in concentrations ranging from those present in PTLs to levels approaching the reported minimum inhibitory concentration (MIC) values. Solutions adjusted to different pH values as well as solutions simulating the chemical composition (nitrites, nitrates, and hydrogen peroxide) in the given PTL (artDC, artAC_air_) were tested in parallel to the tested PTLs. All the solutions were tested on several bacterial strains in various exposure times from 10 min to 24 h.

Gram-negative *Pseudomonas aeruginosa* exhibited greater sensitivity to the tested PTLs and other tested solutions compared to gram-positive *S. aureus*. A complete inhibition of *P. aeruginosa* was achieved after 2 h of exposure to PTL_ACair_, and after 4 h of exposure to PTL_DC_. Chemically prepared alternatives of tested PTLs (referred as artPTL) had no significant effects. The effects of the standard solutions indicated greater resistance to nitrites than to nitrates. The solutions with pH below 5 compromised or fully inhibited the bacterial viability. Thus, acidic environment also proved to have a significant effect on bacterial growth. The higher concentration of nitrates in PTL_ACair_ combined with lower pH (3.5) could explain why this PTL demonstrated better antimicrobial activity than PTL_DC_.

The best antimicrobial effects on gram-positive *S. aureus* were shown by the plasma treated liquid PTL_DC_ (prepared by the inside liquid pinhole discharge supplied by DC high voltage) which reduced the number of colonies by 3 log_10_ after 4 h and by more than 6 log_10_ after 24 h. PTL_ACair_ (prepared by the inside liquid pinhole discharge by AC high voltage with air bubbling) reduced the number of colonies by 3 log_10_, only. Neither the solutions simulating the chemical composition of PTL, nor the standard solutions of nitrites and nitrates had any major inhibitory effect on this bacterial strain. Hydrogen peroxide had shown mild antimicrobial effects. The solutions with different pH values proved that the efficacy of PTL is not dependent on the lower pH, since the pH solutions comparable to the pH of PTLs did not exhibit any inhibition efficacy. However, the combination of lower pH and higher concentration of hydrogen peroxide in combination with other reactive species may have been the reason for increased sensitivity of *Staphylococcus aureus* to PTL_DC_. Enhanced antimicrobial efficacy can be observed particularly over longer exposure times, as indicated by the increased inactivation observed at 24 h. This effect is likely amplified by *S. aureus*’s limited capacity to cope with reactive oxygen species once its catalase-based defences are overwhelmed^52^.

As a part of this study individual long-lived RONS were evaluated to better understand their specific contributions to the antimicrobial efficacy of plasma-treated liquids. By systematically evaluating the antimicrobial effects of hydrogen peroxide, nitrite, and nitrate across a range of concentrations, we were able to demonstrate not only their distinct dose-dependent activity, but also their varying efficacy against different bacterial species. Hydrogen peroxide exhibited bactericidal effects on *S. aureus* only at high concentrations (≥ 10,000 mg∙l^− 1^), yet a partial recovery of viable cells was observed over time, likely due to adaptive stress responses such as catalase upregulation. Similarly, low to moderate concentrations of H_2_O_2_ (50–100 mg∙l^− 1^) led to increased colony counts with increased exposure time. These findings support the well reported concept of the hormetic effect, which is closely linked to the mechanisms of action observed in cold plasma-based treatments, where the biological response depends on the “dose”. Thus, certain reactive species may have either positive or negative effects on the same cells, depending on their concentration. For *P. aeruginosa*, higher sensitivity to all the tested solutions was observed. Interestingly, a high sensitivity to nitrates (4 log_10_ reduction after 2 h of contact with 100 mg∙l^− 1^ nitrates) with no clear recovery trend was observed, indicating less nitrosative stress tolerance. These findings highlight the potential of studying long-lived RONS in isolation as a model to better understand how individual species contribute to microbial inactivation mechanisms, including in contexts beyond plasma-treated liquids. This approach may be particularly valuable for exploration of oxidative and nitrosative stress responses in bacteria, and for deconstruction of the complex chemical environments involved in direct cold atmospheric plasma (CAP) treatment and other redox-based antimicrobial strategies.

The findings of this study support the idea that the antimicrobial efficacy of PTLs is a result of various chemical reactions following the plasma treatment. It is not possible to substitute the PTL by one single aspect of the liquid or by chemical mixing of the major components. While it was not possible to chemically replicate the full composition of the actual PTL, its major species (hydrogen peroxide, nitrites, and nitrates) were tested individually and in combination (artPTLs). However, in a real PTL, various minor components (species) can be introduced either directly from the plasma or formed as a result of the plasma-liquid interaction. These minor (rare) species are more difficult to eliminate by the common adaptation or regulation mechanisms and can therefore exhibit stronger antimicrobial effects. Thus, the efficacy of the PTL is based on the synergistic activity of various reactive species as well as the lowered pH. Lately it has been shown by Seixas et al.^64^ that oxidative stress not only targets DNA, but also results in RNA oxidation. Bacteria, to protect themselves from oxidative stress, induce the expression of several genes (*SoxRS*,* OxyR* and *PerR* ) that confer tolerance to a certain number of free radicals, but high levels of RONS lead to the oxidation of several biomolecules. Notably, RNA is particularly susceptible to this chemical damage leading to translational defects and impaired protein synthesis, particularly through the formation of 8-oxo-G lesions in ribosomal and messenger RNA. These mechanisms offer further insight into why the long-term exposure to RONS-rich PTLs, especially under acidic conditions, results in enhanced antimicrobial activity. Altogether, this supports the idea that bacterial inactivation by PTLs arises not from a single factor, but from multi-target, synergistic interactions among oxidative agents and environmental acidity. This highlights the unique properties of the PTLs making them a promising tool for decontamination processes. In conclusion, both tested PTLs (PTL_DC_ and PTL_ACair_) represent promising agents for decontamination processes thanks to their unique composition and properties acquired in the plasma treatment.

However, the exposure times to attain sufficient inhibition tested in this study are long. The efficacy of the PTLs could be improved by increasing the plasma treatment time. The short treatment time was chosen as a reference for the investigation purpose (to maintain a relatively neutral pH, reasonable quantities of reactive species and operational safety) but could be prolonged if needed. This could potentially be applied in circulatory regime for sterilization of liquids. Various applications that could benefit from this use include the cleansing and maintenance of different heat-sensitive tools or utensils that cannot be autoclaved or sterilized by other heat-based methods. These tools may include different silicone tubes, catheters, endoscopes and others, into which the PTL could be pumped in and clogged inside or circulated through the tube in a continuous regime. The application of PTL for these purposes has been shown in a recent study by Northage et al.^65^where the option to “activate” disinfectant solutions to increase their antimicrobial efficacy by the plasma treatment was also studied.

For the use on animal or human bodies, other effects of the plasma treated liquids need to be considered. In this study we have only focused on the decontamination aspect of the PTL, but there are many other aspects that support the promotion of PTL into the medical world. Various studies have shown an enhancement of wound healing^66^ or perspective cancer treatment^56,67^ as well as improved drug delivery^68^. Furthermore, it is now known that the PTL can be turned into a hydrogel^33,34^which opens a completely new applicational sphere. Given the clinical context of this study - the potential veterinary use, the plasma-treated liquids (mainly turned into the plasma treated hydrogels) offer promising alternatives or adjunctive treatments. The broad-spectrum antimicrobial activity of PTL, particularly at lower-pH and RONS-rich conditions, could be used for topical application in chronic or recurrent *otitis externa*, infected wounds, or surgical site infections. Where the indirect mode of application allows for non‑invasive treatment, which is desirable for animals with sensitive or inflamed tissues. The ability to tailor PTL composition based on dominant microbial species (e.g., hydrogen peroxide‑rich for gram-positives, nitrite-rich for gram-negatives) could pave the way for personalized plasma-based antimicrobial therapies in veterinary dermatology and otology. Therefore, we may conclude that the plasma treated liquids represent an interesting novel tool that may be helpful in veterinary and human medicine.

## Figures and Tables

**Fig. 10:**
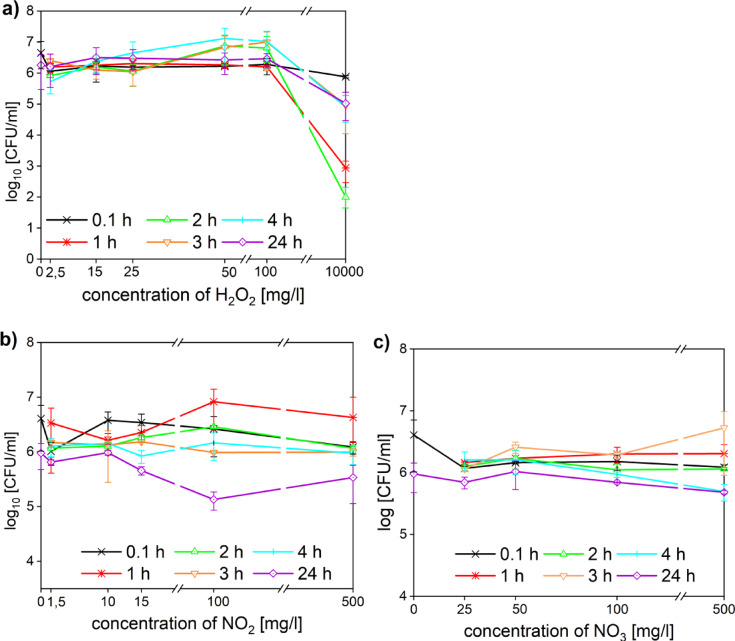
Reduction in *S. aureus* viability using the standard solutions of selected RONS in range of concentrations shown for different exposure times (**a**) hydrogen peroxide (H_2_O_2_,) (**b**) nitrites (NO_2_,) (**c**) nitrates (NO_3_).

## Data Availability

The datasets generated during and/or analyzed during the study are available from the corresponding author on reasonable request.

## Appendix

See Figs. 9 and 10.
